# Comparison of Anterior Ocular Biometric Measurements Using Swept-Source and Time-Domain Optical Coherence Tomography

**DOI:** 10.1155/2020/9739878

**Published:** 2020-09-04

**Authors:** Sisi Chen, Rongrong Gao, Colm McAlinden, Junming Ye, Yiran Wang, Min Chen, Jinhai Huang, Yong Sun, A-Yong Yu

**Affiliations:** ^1^School of Ophthalmology and Optometry and Eye Hospital, Wenzhou Medical University, Wenzhou, Zhejiang, China; ^2^Department of Ophthalmology, Singleton Hospital, Swansea Bay University Health Board, Port Talbot, UK; ^3^Shenzhen Hospital of Integrated Traditional and Western Medicine, Shenzhen, China

## Abstract

**Purpose:**

To compare central corneal thickness (CCT), aqueous depth (AQD), and anterior chamber depth (ACD) measurements using the swept-source (CASIA SS-1000, Tomey, Japan) and time-domain (Visante, Carl Zeiss Meditec, USA) anterior segment optical coherence tomographers (OCT) in normal eyes.

**Methods:**

Sixty-eight eyes of 68 subjects were included. Three consecutive scans of each subject were obtained using both devices in a random order by one experienced operator. Standard deviation (*S*_w_), coefficient of repeatability (CoR), coefficients of variation (CoV), and intraclass correlation coefficients (ICC) were used to evaluate the intraoperator repeatability. Agreement was assessed using the Bland–Altman plots and 95% limits of agreement (LoA).

**Results:**

All measurements of the swept-source OCT (SS-OCT) and time-domain OCT (TD-OCT) showed high repeatability with low CoR (CCT: 2.34 *μ*m and 6.16 *μ*m; AQD: 0.05 mm and 0.09 mm; ACD: 0.06 mm and 0.09 mm), low CoV (CCT: 0.16% and 0.42%; AQD: 0.61% and 0.97%; ACD: 0.53% and 0.83%), and high ICC (>0.98). The mean CCT with SS-OCT was slightly thicker than the results with TD-OCT (difference = 4.55 ± 2.62 *μ*m, *P* < 0.001). There was no statistically significant difference in AQD or ACD measurements between the two devices (0.01 ± 0.05 mm, *P*=0.111; 0.02 ± 0.05 mm, *P*=0.022, respectively). The 95% LoA between the SS-OCT and TD-OCT were −0.59 to 9.69 *μ*m for CCT, −0.10 to 0.12 mm for AQD, and −0.09 to 0.12 mm for ACD.

**Conclusions:**

High levels of repeatability and agreement were found between the two devices for all three parameters, suggesting interchangeability. SS-OCT demonstrated superior repeatability compared with TD-OCT.

## 1. Introduction

Accurate and precise biometry is essential for cataract and refractive surgery planning. Central corneal thickness (CCT) is critical in refractive surgery, which can be useful to reduce the risk of postoperative corneal ectasia, and plays a major role in measuring intraocular pressure (IOP) accurately and diagnosing corneal diseases [1, 2]. Anterior chamber depth (ACD) is the distance from corneal epithelium to anterior surface of crystalline lens, while aqueous depth (AQD) is the distance from corneal endothelium to anterior surface of crystalline lens [3]. ACD and AQD are important parameters that can be used to calculate intraocular lens (IOL) power, select candidates for phakic IOL implantation, and screen for primary angle closure glaucoma [3–5].

Various optical biometers are available to measure these parameters. Optical coherence tomography (OCT) is a noninvasive biometric technique which was first described by Huang et al. [6] and was introduced in clinical practice in 1996 to measure retinal thickness [7]. The first commercially available OCT device designed for anterior segment measurement was a time-domain OCT (TD-OCT). Since then, an increasing number of OCT devices were developed, including the newly designed TD-OCT, spectral-domain OCT (SD-OCT), and swept-source OCT (SS-OCT) [5, 8–10]. The Visante OCT (Carl Zeiss Meditec, Dublin, CA, USA) is a TD-OCT, using a light source of 1310 nm super-luminescent light-emitting diode. The more recent SS-OCT CASIA SS-1000 (Tomey, Nagoya, Japan) uses a 1310 nm swept-source laser. It achieves a higher scan speed of 30,000 A-scans per second, which is 7.5 times that of the Visante TD-OCT, while the axial resolution and transverse resolution are 1.8 times and 2 times [11] that of Visante TD-OCT, respectively [8].

Theoretically, faster measurement speed and higher resolution may result in better precision. In order to prove this hypothesis, the present study sought to comprehensively assess the repeatability of the SS-OCT (CASIA SS-1000) and TD-OCT (Visante) in measuring three anterior segment parameters.

## 2. Subjects and Methods

### 2.1. Subjects

This study included 68 eyes of 68 healthy subjects from the Eye Hospital of Wenzhou Medical University, Zhejiang, China. Patients less than 18 years, or with any corneal opacities, with ocular diseases other than myopia or myopic astigmatism, with contact lens usage (within 4 weeks for rigid contact lens and within 2 weeks for soft contact lens) before examination, and previous ocular surgery were excluded. The study was approved by the Review Board of the Eye Hospital of Wenzhou Medical University and adhered to the tenets of the Declaration of Helsinki.

### 2.2. Examination

Each subject underwent a complete ophthalmic examination on the same day: visual acuity, slit-lamp biomicroscopy, noncontact tonometry, and ophthalmoscopy. One experienced examiner performed three repeated measurements using the CASIA SS-1000 and Visante TD-OCT. The measuring sequence was randomized according to a computer-generated random number sequence to avoid methodological bias. The measurements took place in a dimly-lit room and spent about 20 minutes between 10:00 and 17:00. The patients were asked to sit still and stabilize their heads by using the chin rest and head band. During measurements, the internal fixation targets were used to gain the unaccommodated state.

The CASIA SS-1000 is a swept-source Fourier-domain AS-OCT with a measuring speed of 30,000 axial scans per second [12]. It can measure a 10 mm diameter scanning range. Besides the high axial and transverse resolutions (10 *μ*m and 30 *μ*m), the system also performs large depth scans (6 mm tissue penetration) and a 16 mm × 16 mm horizontal and vertical scan. The built-in software in the CASIA SS-1000 automatically calculates the CCT, AQD, and ACD.

The Visante OCT is the first commercially available time-domain AS-OCT [13]. The scanning speed is 4,000 axial sans per second, and the axial and transverse resolutions are 18 *μ*m and 60 *μ*m, respectively. When the corneal reflex occurs, there appears a vertical white line along the center of the cornea, and then the image is captured. The CCT, AQD, and ACD are calculated semiautomatically using the on-screen calibrated caliper function [14].

### 2.3. Statistical Analysis

Statistical analysis was performed using the SPSS software (version 21.0, SPSS, Inc., Chicago, Illinois, USA). Normal distribution was evaluated using the Kolmogorov–Smirnov test. The results were expressed as mean ± standard deviation. The within-subject standard deviation (*S*_w_), coefficient of repeatability (CoR, 2.77*S*_w_, namely, reproducibility limit), coefficients of variation (CoV, *S*_w_/mean), and intraclass correlation coefficients (ICC) were used to evaluate the repeatability of the two AS-OCT devices [15, 16]. In addition, lower values for CoR and CoV represent better repeatability. The ICC is centered and scaled using a pooled mean and standard deviation, with values ranging from 0 to 1 (<0.75 = poor correlation, 0.75 to <0.90 = moderate correlation, ≥0.90 = high correlation). Agreement between the two instruments was analyzed with Bland–Altman plots and 95% limits of agreement (95% LoA, mean difference ± 1.96SD). The narrower the 95% LoA, the better the agreement [17]. *P* values <0.05 were considered statistically significant.

## 3. Results

This study involved 68 right eyes from 68 healthy volunteers (37 females and 31 males), with a mean age of 25.56 ± 2.07 years (range: 21 to 35 years). The mean spherical equivalent refraction was −4.34 ± 2.25 D (range: −0.50 to −10.00 D).  Table 1 displays the mean values of the parameters as well as the intraobserver repeatability outcomes. The CCT, AQD, and ACD values showed high intraobserver repeatability for both instruments, with small values for CoR and with CoV values less than 0.97% for all parameters. ICC values were greater than 0.98 for both devices. The CoR and CoV values for CCT, AQD, and ACD measured by TD-OCT were more than 2.6 times, 1.5 times, and 1.5 times that of SS-OCT, respectively, indicating better repeatability with the SS-OCT for all three parameters.

**Figure 1:**
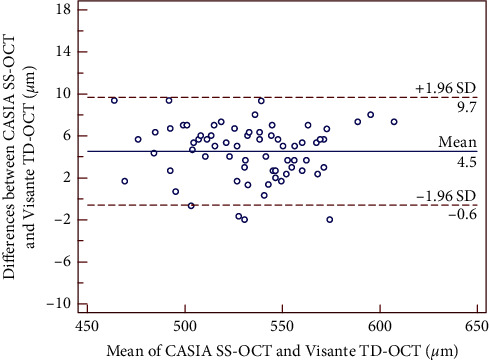
Bland–Altman plot showing the agreement for CCT measurements between the CASIA SS-OCT and the Visante TD-OCT. The solid line indicates the mean difference (bias), and the dotted lines indicate 95% limits of agreement (LoA).

**Figure 2:**
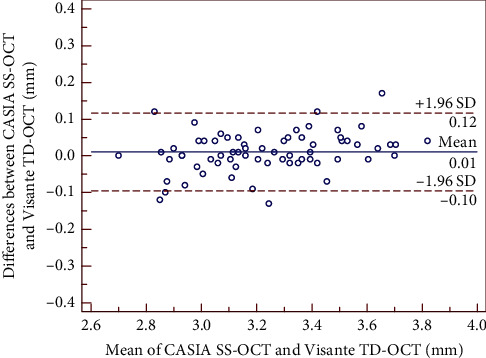
Bland–Altman plot showing the agreement for AQD (corneal endothelium to the anterior lens) measurements between the CASIA SS-OCT and the Visante TD-OCT. The solid line indicates the mean difference (bias), and the dotted lines indicate 95% limits of agreement (LoA).

**Figure 3:**
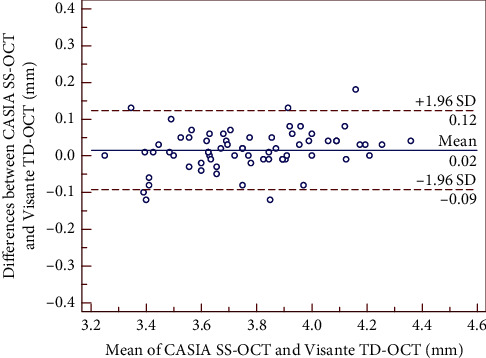
Bland–Altman plot showing the agreement for ACD (from the corneal epithelium to the anterior lens) measurements between the CASIA SS-OCT and the Visante TD-OCT. The solid line indicates the mean difference (bias), and the dotted lines indicate 95% limits of agreement (LoA).

The mean differences between SS-OCT and TD-OCT are shown in Table 2. There was no significant difference in the mean AQD and ACD measurements, whereas the CCT value showed a statistically significant difference (*P* < 0.01), but it was clinically insignificant with narrow 95% LoA. Bland–Altman plots showed high agreements between the two devices. The 95% LoA for CCT, AQD, and ACD were −0.6 to 9.7 *μ*m, −0.10 to 0.12 mm, and −0.09 to 0.12 mm, respectively, as shown in Figures 1–3.

## 4. Discussion

This study found significant differences in CCT between SS-OCT and TD-OCT, but the difference was low (4.55 ± 2.62 *μ*m) and within a clinically acceptable range. Several reasons may be attributable to this difference. Firstly, SS-OCT is a Fourier-domain OCT that has higher sensitivity and faster measurement speed than TD-OCT [18, 19]. Secondly, the two devices use different refractive indices to calculate anterior segment dimensions. The cornea refractive index was 1.376 for SS-OCT and 1.388 for TD-OCT, whereas the aqueous humor refractive index was 1.336 for SS-OCT and 1.343 for TD-OCT [11]. Thirdly, the TD-OCT places the anterior corneal surface boundary slightly below the anterior corneal surface, so the current study manually located the corneal surface using the caliper function on the instrument; however, the SS-OCT automatically measures the CCT, which may also cause the difference [20].

Zhang et al. [21] recently assessed the measurements of AQD using both devices and reported excellent repeatability (CASIA SS-1000, CoR = 0.099 mm, ICC = 0.946; Visante TD-OCT, CoR = 0.105 mm, ICC = 0.980); however, they investigated 97 eyes from 49 myopia patients; binocular correlation may have some influence on the results. On the contrary, we found the SS-OCT had better repeatability. The CoR and CoV values for CCT, AQD, and ACD measured with TD-OCT were more than 2.6 times, 1.5 times, and 1.5 times that of SS-OCT, respectively; the ICCs of both AS-OCT devices were higher than their results (CCT, ICC = 0.999 and 0.995; AQD, ICC = 0.995 and 0.985; ACD, ICC = 0.994 and 0.984). Sabatino compared two SS-OCT devices (IOLMaster 700, Carl Zeiss Meditec AG and Argos, Movu, Inc.), and both devices showed high ICCs in measuring CCT (>0.988) and ACD (>0.999) [22]. Hua et al. [23] found the SS-OCT using a wavelength of 1060 nm (OA-2000, Tomey, Japan) provided highly repeatable measurements of CCT and ACD, with CoVs less than 0.668% and 0.426%, CoRs less than 14.06 *μ*m and 0.057 mm, and ICCs greater than 0.991 and 0.999, respectively. Other studies found that the repeatability of the CASIA SS-1000 was also high in postkeratoplasy and keratoconus cases (ICC >0.970) [12, 24]. Our results for the repeatability with Visante TD-OCT were marginally better than previous studies [13, 25, 26]. Nemeth et al. [26] reported the CoV of ACD measurement was less than 1.9%; Li et al. [25] reported 13.57 *μ*m for CoR, 0.90% for CoV, and 0.98 for ICC in measuring CCT by TD-OCT, which was similar to the results reported by Huang et al. (ICC = 0.989) [13]. These studies mentioned above provide indirect evidence that the repeatability of SS-OCT was better than TD-OCT.

When compared with ultrasound pachymetry, studies found the CASIA SS-1000 and Visante TD-OCT underestimated CCT by 8.9 *μ*m and 16.5 *μ*m, respectively [27, 28]. It has also been reported that the CASIA SS-1000 and Visante TD-OCT measured lower CCT values (12.46 *μ*m and 17.79 *μ*m, respectively) than Scheimpflug devices in normal eyes [24, 29]. Theories to explain the lower values obtained by AS-OCT may be that both AS-OCT devices measured CCT along the optical axis, whilst ultrasound pachymetry measurements may be affected by tilt and decentration [30]. In addition, unlike Scheimpflug devices, OCT does not include tear film thickness when measuring pachymetry [31]. The difference between the two AS-OCT devices and ultrasound pachymetry or Scheimpflug imaging indirectly demonstrated that SS-OCT tended to give higher readings than TD-OCT, which agrees with the outcomes of the present study.

The Bland–Altman plots and 95% LoA have been used to assess the level of agreement between AS-OCT devices and other instruments in the measurement of CCT. It was reported that the 95% LoA was −20.3 to 5.9 *μ*m between CASIA SS-1000 and ultrasound pachymetry [32], which was narrower compared to the results between Visante TD-OCT and ultrasound pachymetry (−6.1 to 39.1 *μ*m) [28]. The 95% LoA between CASIA SS-1000 or Visante TD-OCT and Scheimflug devices were also poor, with maximum values more than 35 *μ*m [24, 33, 34]. Earlier studies reported that the 95% LoA between SS-OCT and OLCR was good, with a maximum value of 22.04 *μ*m in normal eyes (OA-2000), [35] and 15.7 to 16.9 *μ*m in cataractous eyes (IOLMaster 700) [36, 37]. Cruysberg et al. [38] reported acceptable but a wider 95% LoA range between OLCR and the Visante TD-OCT (24.1 to −1.9 *μ*m) in healthy volunteers. With respect to the agreement between the CASIA SS-OCT and Visante TD-OCT, our current study revealed narrow 95% LoA, −0.59 to 9.69 *μ*m, which was better than a previous study (maximum value about 15 *μ*m), [11] indicating that the two devices can be used interchangeably for CCT measurement in clinical practice.

In terms of AQD and ACD, it is known that accommodation may change the anterior surface of the lens, causing a decrease of AQD and ACD [39]. In order to minimize the influence of accommodation, both devices used an internal fixation target. Few studies have compared the SS-OCT with TD-OCT in measuring AQD [11, 40, 41]. Interestingly, Aptel et al. [11] reported that the AQD measured with the CASIA SS-1000 was significantly larger than that with the Visante TD-OCT in normal eyes (mean difference 0.12 ± 0.08 mm), and the LoA range was larger than 0.25 mm. The current study found a smaller mean difference in AQD (0.01 ± 0.05 mm) and ACD (0.02 ± 0.05 mm) between the devices and better agreement with narrower 95% LoA (−0.10 to 0.12 mm and −0.09 to 0.12 mm, respectively). It is known that AQD and ACD are inheritable traits that are affected by age, sex, and race [42]. The former study was performed in France, with patients' mean age of 38.6 ± 15.1 years. By contrast, younger Chinese healthy subjects were included in our study. These differences may contribute to the discrepancy. Angmo et al. [40] compared measurements using the same two AS-OCT devices in patients with primary angle closure. The mean difference for AQD was 0.0112 ± 0.106 mm and the 95% LoA was quite narrow, +0.22 to −0.20 mm. Chansangpetch et al. [43] compared a different SS-OCT device (CASIA 2, Tomey Corporation, Nagoya, Japan) with the Visante TD-OCT in 53 eyes (age range: 52 to 86 years) and found that the agreement was excellent in measuring AQD between both devices (ICC, 0.992; LoA, −0.057 to 0.093 mm).

The main limitation of the current study is that we only evaluated anterior segment measurements in young, myopic population; further research in older population, or eyes with ocular disease such as cataract, is warranted. Moreover, we did not compare the results with other OCT biometry, including SD-OCT or similar instruments based on TD-OCT and SS-OCT.

In summary, both SS-OCT and TD-OCT devices provided excellent repeatable measurements of CCT, AQD, and ACD. However, the repeatability was better with SS-OCT. Significant differences were found between the two devices for the measurement of CCT, but the difference was small enough to be considered clinically insignificant. Good agreement in terms of CCT, AQD, and ACD was found, signifying that the two devices can be used interchangeably in normal eyes.

## Figures and Tables

**Table 1:** Intraobserver repeatability outcomes provided by SS-OCT and TD-OCT devices.

Parameter	Observer	Mean ± SD	*S *_w_	CoR	CoV (%)	ICC (95% CI)

CCT (*μ*m)	SS-OCT	535.59 ± 30.31	0.85	2.34	0.16	0.999 (0.999 to 0.999)
	TD-OCT	531.04 ± 30.46	2.22	6.16	0.42	0.995 (0.992 to 0.997)
AQD (mm)	SS-OCT	3.24 ± 0.27	0.02	0.05	0.61	0.995 (0.992 to 0.996)
	TD-OCT	3.23 ± 0.25	0.03	0.09	0.97	0.985 (0.977 to 0.990)
ACD (mm)	SS-OCT	3.78 ± 0.26	0.02	0.06	0.53	0.994 (0.991 to 0.996)
	TD-OCT	3.76 ± 0.25	0.03	0.09	0.83	0.984 (0.977 to 0.990)

SD = standard deviation; *S*_w_ = within-subject standard deviation; CoR = coefficient of repeatability (2.77 *S*_w_); CoV = within-subject coefficient of variation; ICC = intraclass correlation coefficient; CCT = central corneal thickness; AQD = aqueous depth; ACD = anterior chamber depth.

**Table 2:** The mean difference, paired *t* test (*P* value), and 95% LoA for differences between the SS-OCT and TD-OCT devices.

Device pairings	Mean ± SD	*P* value	95% LoA

CCT (*μ*m)	4.55 ± 2.62	<0.001	−0.59 to 9.69
AQD (mm)	0.01 ± 0.05	0.111	−0.10 to 0.12
ACD (mm)	0.02 ± 0.05	0.022	−0.09 to 0.12

SD = standard deviation; CCT = central corneal thickness; AQD = aqueous depth; ACD = anterior chamber depth.

## Data Availability

The data used to support the findings of this study are available from the corresponding author upon request.
